# Telehealth Can Be Implemented Across a Musculoskeletal Service Line Without Compromising Patient Satisfaction

**DOI:** 10.1177/1556331620977171

**Published:** 2021-02-21

**Authors:** Paul T. Greenfield, Wesley J. Manz, Emily L. DeMaio, Sage H. Duddleston, John W. Xerogeanes, T. Scott Maughon, Corey C. Spencer, Alexander Dawes, Scott D. Boden, Kyle E. Hammond, Eric R. Wagner, Michael B. Gottschalk, Charles A. Daly, Mathew W. Pombo

**Affiliations:** 1 Department of Orthopaedic Surgery, Emory University, Atlanta, GA, USA

**Keywords:** telemedicine, orthopedic surgery, sports, practice specialty, upper extremity, body sites, COVID-19

## Abstract

*Background:* The COVID-19 pandemic has led to changes to in-office orthopedic care, with a rapid shift to telemedicine. Institutions’ lack of established infrastructure for these types of visits has posed challenges requiring attention to confidentiality, safety, and patient satisfaction. *Purpose*: The aim of this study was to analyze the feasibility of telemedicine in orthopedics during the pandemic and its effect on efficiency and patient satisfaction. *Methods*: Patients seen by the Emory University Department of Orthopaedics Sports Medicine and Upper Extremity Divisions via telemedicine from March 23 to April 24, 2020, were contacted by telephone. Each patient was asked to respond to questions on satisfaction, ease of use, and potential future use; satisfaction with telemedicine and previous clinical visits were measured using a modified 5-point Likert scale. *Results*: Of the 762 patients seen, 346 (45.4%) completed the telemedicine questionnaire. Satisfaction varied by visit type, with average scores of 4.88/5 for in-office clinic visits versus 4.61/5 for telemedicine visits. There was no significant difference among age groups for satisfaction ratings. Patients 65 years old or older reported significantly longer visit times and decreased ease of use with the telemedicine platform. *Conclusion*: Telemedicine in a large orthopedics department was successfully implemented without compromising patient satisfaction. The use of telemedicine allows many patients to be seen quickly and efficiently without diminishing their musculoskeletal clinical experience.

## Introduction

Certain economic, physical, and social barriers exist that interfere with health care providers’ abilities to provide adequate care to patients in an office setting. These include, but are not limited to, the financial burden of the office visit, geographic proximity to specialty providers, the availability of public transit, and the time required to attend a visit [1,16,20,37]. Traditional in-office orthopedic care has struggled to address these well-documented, predictable barriers. Despite technological expansion in many fields, there remains little use of virtual visits in urban areas within orthopedic surgery [13,21]. The outbreak of coronavirus 2 (SARS-CoV-2) that resulted in the coronavirus disease 2019 (COVID-19) pandemic has forced the need for rapid implementation of telemedicine and exposed the lacking virtual visit infrastructure of many institutions [23].

Telemedicine, which involves utilizing technology to remotely diagnose and treat patients, has shown promise for multiple applications within orthopedics [2,3,7,12,15,28,29]. These include postoperative care, rehabilitation, and remote consultation [4-6,19,27,30,32-34,36]. The ability of providers to treat patients virtually provides opportunities for improving efficiency without sacrificing patient satisfaction [10,18,19]. In an ideal setting, slow implementation of a department-wide telemedicine program would allow for front-end troubleshooting and small-scale optimization [24]. However, the crisis resulting from the pandemic provided a uniquely challenging task necessitating rapid deployment of a telehealth platform in a large hospital system [23]. Functionality, confidentiality, and patient safety are critical in ensuring the success of such a program [11,26]. These factors must be delicately balanced with patient and provider satisfaction [40]. Although it is difficult to measure the inherent safety of such an intervention, quantifying provider and patient experiences is relatively straightforward [17,25].

Analyzing data from the current outbreak offers a short-term glimpse into the utilization of telemedicine in orthopedics [23,38]. Ultimately, telemedicine could be used as a permanent tool during a non-pandemic period if the restrictions around its use are lifted [12,28]. Despite recent literature highlighting the viability of telemedicine in certain niches within orthopedics, its widespread adoption and implementation has yet to occur [16,31]. Critical analysis of the rapid setup, implementation, and deployment of telemedicine platforms during times where in-clinic visits are either not permitted or are discouraged may provide further insight into the necessity of a permanent telemedicine practice within orthopedics. Even at a greatly reduced patient load, the establishment of permanent telemedicine practices has the potential to enhance our patient care and consolidate orthopedic clinic clutter [14]. In addition, a permanent telehealth platform may offer an alternative to those whose access to clinical care is constrained by socioeconomic factors [4,9].

There remains a paucity of studies examining the rapid setup and implementation of telemedicine, as well as impact on patient experience. The purpose of this study was to determine the feasibility of rapidly implementing a department-wide telemedicine platform and its effect on efficiency and patient satisfaction within 2 divisions, Sports Medicine and Upper Extremity. The primary objective was to compare patient satisfaction of the newly implemented telehealth system with that of recent in-office visits prior to the emergence of the pandemic. The secondary objectives were to compare the time burden of telehealth and in-person visits on the patient, and to compare telehealth experience across age groups. We hypothesized that the majority of patients would be willing to be seen virtually, while the satisfaction would be comparable to in-office visits. We hoped such analysis would be useful not only as a model amid the current pandemic but potentially lend itself toward construction of a future permanent telehealth infrastructure in orthopedics.

## Materials and Methods

The Emory Department of Orthopaedics saw patients via telemedicine visits from March 23 to April 24, 2020, secondary to the COVID-19 pandemic. During this period, patients seen by surgeons within the Divisions of Upper Extremity and Sports Medicine were contacted as a part of a quality improvement initiative to ensure that patient needs were being met during this unprecedented time. This study is a retrospective analysis of the telemedicine quality improvement initiative. Institutional Review Board (IRB) approval was obtained to use this information for research purposes and publication.

All patients had telemedicine visits with 1 of the 7 surgeons in the Upper Extremity or Sports Medicine divisions during the aforementioned time period. Following their visit, patients were contacted via telephone by one of the study authors and asked to complete our telemedicine survey. Their responses were recorded and stored in a password-protected, encrypted database.

The inclusion criteria required patients to be over the age of 18 years. Patients must have undergone a telemedicine visit during the period of interest utilizing a computer, tablet, and/or smartphone. Included patients may or may not have undergone prior surgical intervention. The exclusion criteria included patients who were seen solely in the office for a medically necessary visit during the pandemic. In-person visits were at the discretion of the attending physicians and included patients with time-sensitive pathology such as a recent surgery, acute trauma, or possible infection. In addition, patients who declined to participate or who were unable to be successfully contacted were excluded. Furthermore, any patients who did not speak English as their primary language were excluded due to limited access to interpreter services. All eligible patients were contacted on 2 separate attempts prior to being deemed unable to contact.

**Fig. 1.**
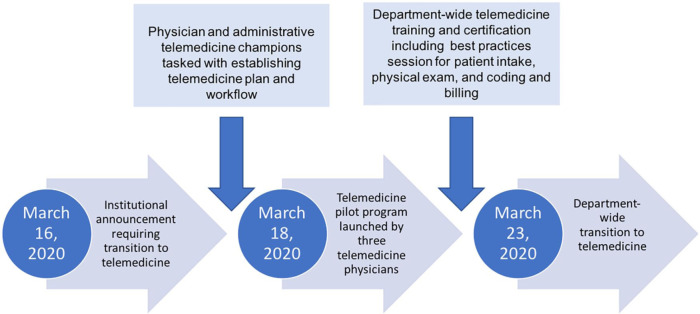
This flowchart demonstrates our implementation timeline.

On March 16, 2020, an institutional announcement was made directing all physicians to transition to remote working via telemedicine. During this time frame, a physician and administrative telemedicine champion were tasked to establish a workflow for patient care using telemedicine. Two days later on March 18, 2020, a small pilot program was tested with 3 different musculoskeletal physicians. Prior to operationalizing the telemedicine process, a department-wide telemedicine workflow training and certification was done. This included educating providers and their teams about the technology, changing clinic templates, working with administrative assistants to provide secure links to patients, testing advanced features of several telemedicine platforms, and developing best practices for patient intake, physical examination, and telemedicine coding and billing. Within 7 days, on March 23, 2020, the department was transitioned to a complete telemedicine clinical model (Fig. 1). Patients were seen by the senior authors from remote locations, with patients located in the same state as the provider’s medical licensure allowed. The Department of Orthopaedics utilized Zoom Enterprise (Zoom Video Communications, Inc, San Jose, CA), a Health Insurance Portability and Accountability Act (HIPAA)-compliant video conferencing application, to conduct visits.

**Fig. 2.**
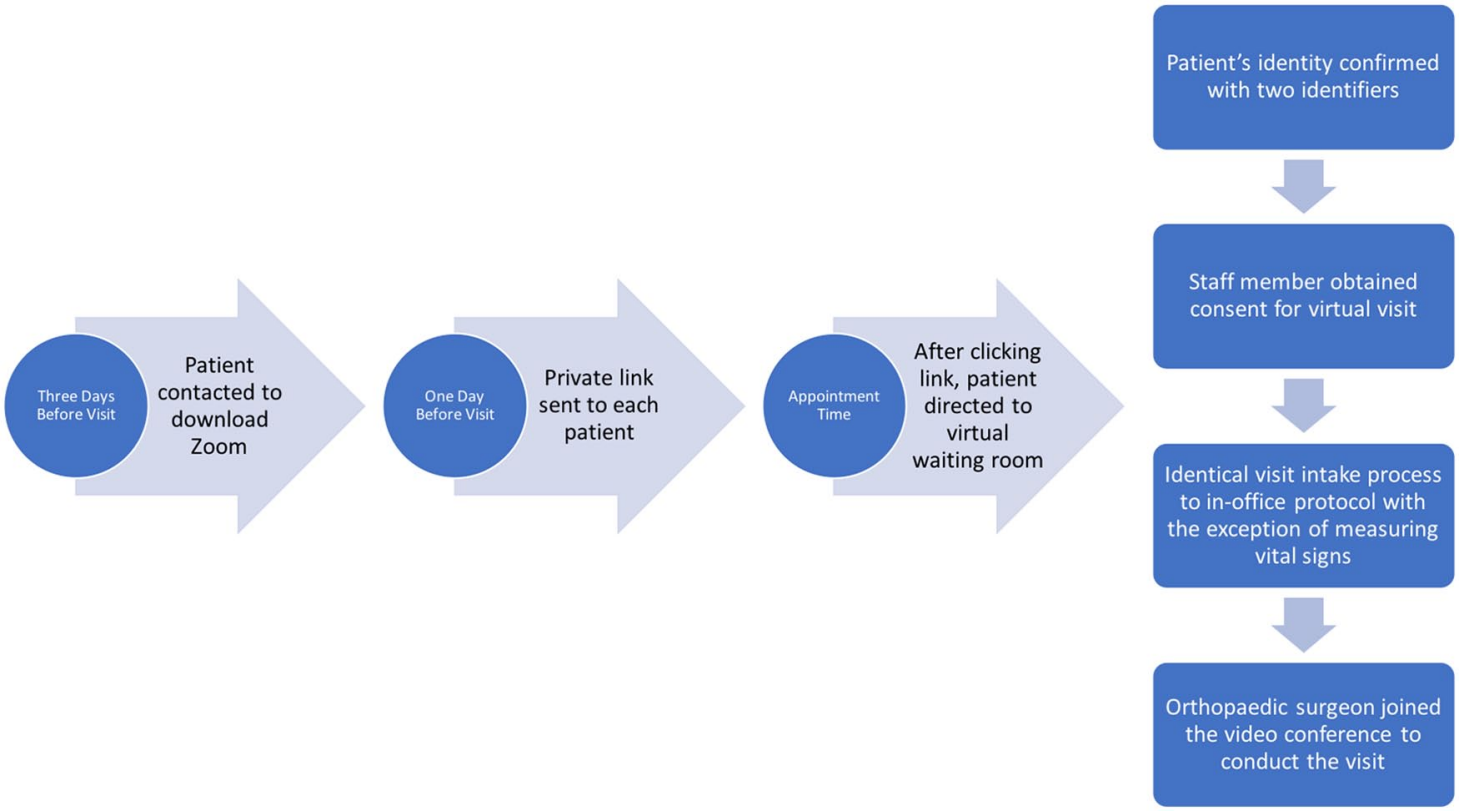
This diagram displays the steps leading up to and during each telemedicine visit.

In the days leading up to their visit, patients were contacted with instructions on downloading Zoom to their Internet-enabled device (ie, computer, tablet, smartphone, etc). Prior to the scheduled telemedicine visit, a private link was sent to each patient that was then accessed at the time of their scheduled visit. The Zoom link directed the patients into the physician’s virtual “waiting room.” Once in the waiting room, the patient’s identity was confirmed using 2 identifiers. Afterward a staff member obtained consent to perform a telemedicine visit and proceeded to complete their visit intake. The intake process was identical to the in-office visits, excluding measuring vital signs. Following the intake process, the orthopedic surgeon joined the video conference to conduct the visit. The physician led the visit in an identical manner to an in-person appointment with the sole difference of utilizing a visualization-based physical exam rather than physically examining the patient [38]. At the conclusion of the visit the orthopedic surgeon ended the Zoom call, thereby ending the encounter (Fig. 2). In select instances, patients and/or clinical staff encountered technical difficulties with Zoom, preventing the appointment to be carried out as scheduled. In these cases, patients were seen over FaceTime (Apple Inc, Cupertino, CA) and Doximity Dialer Video Call (Doximity Inc, San Francisco, CA) if they had a device capable of using this software. Phone calls were utilized as a last resort for those patients who did not have access to the Internet. Any patient deemed emergent (fractures, septic joints, etc) was set up for an appropriate in-office visit or referred to the emergency department accordingly, especially if timely radiographic examination was necessary for clinical decision making.

The telemedicine questionnaire was designed by clinical staff through a quality improvement initiative to quantify patients’ satisfaction with the telemedicine visit. (Supplemental Appendix A) Satisfaction was gauged by utilizing a Likert scale from 1 to 5, with 1 being extremely dissatisfied and 5 being extremely satisfied [19]. Furthermore, there were questions that quantified efficiency, such as patient perceived visit duration and ease-of-use of the Zoom platform. In addition, we analyzed provider-specific factors that inherently affect the quality of the visit, such as answering patient questions and explaining treatment plans. In addition, patients were specifically asked if a physical examination over telemedicine detracted from their appointment or made them feel uncomfortable. To create a clinical control, patients participating in our telemedicine questionnaire were asked about their last in-office visit within the last 2 years. These questions on visit satisfaction used a Likert scale and asked patients to estimate the perceived amount of total time taken to travel to and conduct their in-office visit. Patient perceived satisfaction and visit time for both encounters were compared.

Although the majority of orthopedics visits were transitioned to telemedicine, 1 provider was available for patients that required in-office emergent visits throughout the study time period. A protocol was developed for staffing of the musculoskeletal center in an effort to protect providers and patients while preventing interruptions in necessary care [35]. This task was accomplished by dividing all staff and providers into 2 teams cycling between “active-duty” and “working remotely” every 2 weeks in light of the viral incubation period and with strict adherence to team assignments and preventing exposure while “working remotely” [35]. The providers on “active-duty” were available to see patients that were deemed “medically necessary” as previously described, especially if timely radiographic or in person physical examination was necessary for clinical decision making or if they needed a procedure, which included, but was not limited to injections, splinting, removal of sutures, etc [35]. Barring the aforementioned necessities for in person visits, all other “working remotely” physicians and “active duty” physicians not in office were providing care through telemedicine.

Records were analyzed from the Billing Department to assess the number of patients seen during the time period of interest and compared with the historical volume of the entire musculoskeletal center during the same time period in 2019. Patients were able to schedule telemedicine visits through standard patient access processes or by calling the provider’s scheduling team directly. Any patients who were due for follow-up appointments but were not yet scheduled were contacted by clinical staff to schedule telemedicine visits. The volume data represents all patient visits across the enterprise’s musculoskeletal service line including physical medicine and rehabilitation, sports medicine, physiatry, orthopedics, and spine.

Descriptive statistics were used to describe patient demographic information. Differences in categorical variables were assessed using the χ^2^ or Fisher’s exact test. Paired samples *t* tests were used to compare parametric continuous data between 2 groups and 1-way analysis of variance (ANOVA) with post hoc comparisons were used for comparisons between 3 groups. All tests were 2-sided, and *P* value < .05 was considered statistically significant. All analyses were performed with SPSS software version 25.0 (IBM Corporation, Armonk, NY).

## Results

Of the 762 patients seen by the Divisions of Sports Medicine and Upper Extremity during the period of interest, 346 (45.4%) patients were contacted and completed the Telemedicine Questionnaire. The average patient age was 52.4 years old (range: 18 to 88 years). Our population included 183 women (52.9%) and 163 men (47.1%) (Table 1). The musculoskeletal service line, representing 80 full-time equivalent providers, conducted 8,242 telemedicine visits over this period (Table 2).

**Table 1.** Patient demographics.

Total patients	346
Average age, (*SD*)	52.4 (17.3)
Age group	
18-49	129 (37.3)
50-64	115 (33.2)
65+	102 (29.5)
Sex	
Male	163 (47.1)
Female	183 (52.9)
Department	
Upper extremity	166 (48.0)
Sports medicine	180 (52.0)
Affected body region	
Upper limb	235 (67.9)
Lower limb	108 (31.2)
Other^a^	3 (0.9)
Telemedicine visit type	
Zoom	307 (88.7)
FaceTime	17 (4.9)
Phone call	22 (6.4)

Presented as n (% of total).

a Includes 2 back and 1 sternum.

**Table 2.** Clinical volume comparison.

Date	Pre-pandemic volume	Date	COVID-19 volume^a^
Week of March 17, 2019	3,449	Week of March 15, 2020	753 (21.8)
Week of March 23, 2019	3,316	Week of March 22, 2020	969 (29.2)
Week of March 31, 2019	2,653	Week of March 29, 2020	1,242 (46.8)
Week of April 7, 2019	3,418	Week of April 05, 2020	1,416 (41.4)
Week of April 14, 2019	3,233	Week of April 12, 2020	1,985 (61.4)
Week of April 21, 2019	3,273	Week of April 19, 2020	1,877 (57.3)
Grand total	19,333	Grand total	8,242 (42.6)

a Presented as n (% of prior year volume).

Satisfaction scores varied by visit type, with a mean satisfaction for in-office clinic visits of 4.88 versus telemedicine visits mean satisfaction of 4.61 (*P <* .001) (Table 3). In addition, satisfaction did not change over the 4 week study period (Table 4). For patients undergoing telemedicine visits, satisfaction was assessed by age group with those aged 18 to 49 years having an average of 4.61 compared with 50- to 64-year-olds with 4.58 and those older than 65 years old with 4.65 (*P* = .795) (Table 5). In-office clinic visits were an average of 96.5 minutes including travel time as compared with 20.0 minutes for telemedicine (*P <* .001). By age group, the mean telemedicine durations for patients 18 to 49, 50 to 64, and those greater than 65 years old were 18.5, 19.9, and 22.0 minutes, respectively (*P* = .042) (Table 5). Telemedicine duration was also analyzed by body region with upper limb injuries having an average time of 21.3 minutes compared with lower limb with 17.3 (*P* = .001) (Table 6). Duration of clinic visit results were not correlated with satisfaction (Pearson correlation coefficient of −0.023, *P* = .653).

**Table 3.** Comparison of time and satisfaction between clinic and telemedicine visit.

	Visit type		Difference	*P* value
	Clinic^a^	Telemedicine		
Satisfaction^b^	4.88 (0.40)	4.61 (0.76)	0.27	<.001
Time^c^	96.49 (55.79)	20.02 (10.82)	76.47	<.001

a Includes travel time.

b Average (*SD*).

c Average in minutes (*SD*).

**Table 4.** Time and satisfaction between visit type by week.

	n (%)	Visit type		Difference	*P* value
		Clinic	Telemedicine		
Satisfaction^a^					
Week of March 22, 2020	59 (17.1)	4.88 (0.38)	4.56 (0.84)	0.32	.008
Week of March 29, 2020	67 (19.4)	4.94 (0.24)	4.60 (0.81)	0.34	.001
Week of April 5, 2020	78 (22.5)	4.84 (0.55)	4.69 (0.55)	0.15	.030
Week of April 12, 2020	87 (25.1)	4.89 (0.39)	4.61 (0.82)	0.28	.004
Week of April 19, 2020	55 (15.9)	4.84 (0.36)	4.57 (0.76)	0.27	.011
Time^b^					
Week of March 22, 2020	59 (17.1)	110.85 (70.57)	19.69 (11.77)	91.15	<.001
Week of March 29, 2020	67 (19.4)	96.57 (52.91)	19.43 (10.89)	77.14	<.001
Week of April 5, 2020	78 (22.5)	92.76 (55.20)	21.59 (10.82)	71.17	<.001
Week of April 12, 2020	87 (25.1)	95.29 (47.97)	21.21 (10.97)	74.08	<.001
Week of April 19, 2020	55 (15.9)	88.18 (52.77)	16.96 (8.98)	71.22	<.001

a Average (*SD*).

b Average in minutes (*SD*).

**Table 5.** Telemedicine analysis by age group.

	Age groups			*P* value*
	18-49	50-64	65+	
Total patients	129	115	102	
Time,^a^ minutes	18.47 (9.2)	19.94 (10.8)	22.07 (12.4)	**.042**
Mean difference	0	1.47	3.60	
*P* value**	—	.534	**.032**	
Satisfaction^a^	4.61 (0.72)	4.58 (0.81)	4.65 (0.75)	.795
Mean difference	0	–0.03	0.04	
*P* value**	—	.962	.902	
Ease of use^a^	4.84 (0.48)	4.68 (0.78)	4.51 (0.98)	**.004**
Mean difference	0	–0.16	–0.33	
*P* value**	—	.235	**.003**	
Required assistance^b^	6 (4.7)	17 (14.8)	21 (20.6)	**.007**
Future use?^b^				
Yes	117 (90.7)	96 (83.5)	81 (79.4)	
No	8 (6.2)	10 (8.7)	14 (13.7)	.132
NP	4 (3.1)	9 (7.8)	7 (6.9)	

Significant *P* values bolded.

*NP* no preference.

a Average (*SD*).

b Presented as n (% of age group).

* Univariate comparison. **P value compared with 18 to 49 age group.

**Table 6.** Telemedicine analysis by body region.

	Body region		Difference	*P* value
	Upper limb	Lower limb		
Total	235	108		
Time, minutes	**21.29 (11.35)**	**17.26 (9.20)**	**4.03**	**.001**
Satisfaction	4.62 (0.77)	4.59 (0.73)	0.03	.745
Ease	4.66 (0.83)	4.75 (0.61)	–0.09	.300
Physical exam	4.44 (0.93)	4.25 (1.10)	0.19	.102

Presented as averages (*SD*); significant values bolded.

Additional details of telemedicine analysis by age group and body region, including satisfaction, ease of use, and willingness to use again, can be found in Tables 5 and 6, respectively. Finally, subgroup analysis of 91 patients establishing new patient care via telemedicine visits yielded a patient satisfaction average of 4.74 and mean visit duration of 23.4 minutes, 3.4 minutes longer than established patients (*P =* .010) (Table 7).

**Table 7.** Comparison of time and satisfaction between telemedicine patient type.

	Patient type		Difference	*P* value
	New	Established		
Total patients	91	346		
Satisfaction^a^	4.74 (0.61)	4.61 (0.76)	0.13	.151
Time^b^	23.42 (12.26)	20.02 (10.82)	3.40	.010

a Average (*SD*).

b Average in minutes (*SD*).

## Discussion

We sought to assess the feasibility of a rapid transition to telemedicine in a large and comprehensive musculoskeletal care practice and assess the overall patient experience within the divisions of Upper Extremity and Sports Medicine during an accelerated introduction of a new telemedicine platform. We hypothesized that patient satisfaction would be comparable to that of in-office visits and that a majority of patients would be willing to be seen virtually. We demonstrated the successful implementation of a telehealth care delivery model across a large, multifaceted musculoskeletal service line. Time-burden was notably decreased for patients, and our orthopedic department was able to achieve a teleclinic volume of 61.4% of normal by the fifth week of implementation. Despite showing an overall patient-reported satisfaction to be slightly less for telemedicine visits than clinical visits during the same time, the magnitude by which satisfaction decreased was minimal and likely not clinically relevant. Furthermore, we have anecdotal reports that some providers were receiving higher patient satisfaction scores through telemedicine visits versus their historical in-office scores. In addition, within our chosen cohort of sports medicine and upper extremity patients, the historical data for these sections’ patient satisfaction scores are routinely the highest within the department. Patient satisfaction scores included the early experience of implementation prior to refinement of protocols that made for a more seamless experience for patients and staff, but when analyzed separately, these early weeks compared favorably to the later experience.

There are certain limitations inherent to the novelty and circumstances surrounding this study (ie, the COVID-19 pandemic). Thus, interpretation of data must be taken in context and may have confounding effects not recognizable for years to come. However, due to the momentum of telemedicine implementation, it is unlikely that its use will drop to pre-pandemic levels, and we must utilize data to inform decisions as we move toward designing processes and allocating resources for the next stage as global health care providers. Furthermore, recall bias is inherently present as all responses were recorded retrospectively over the phone. The use of video telehealth products requires access to a smartphone or computer not available to all patients. In addition, our department did not have translator capabilities for telemedicine calls, further limiting access to care and the generalizability of the study. Although a certain level of competency is required to use a telemedicine platform, our study included all patients, even those who experienced technical difficulties. Certain patients, particularly the elderly, might be less comfortable with these technologies and may decline a telemedicine visit for non-emergent musculoskeletal issues although this was generally not the case with our patients as we had staff to troubleshoot technical difficulties before the visit. In addition, while COVID-19 has provided a broad implementation of telemedicine for patients with musculoskeletal ailments, it is fair to assume there may be lowered expectations for these visits since there was no in-person visits available for non-urgent complaints, thereby falsely elevating patient satisfaction levels. In our study, we did not further separate “Established” patients into subgroups including postoperative and non-postoperative patients, thereby limiting comparison of results with prior studies. This study took place in a large academic musculoskeletal service line, and the infrastructure of our program may not lend itself to other institutions.

Of the 63.1% of accredited orthopedic residency programs reported to have a working telemedicine program, 86% were initiated at the onset of governmental restrictions following COVID-19 [31]. To date, only Loeb et al has addressed considerations for rapidly applying telemedicine in a large orthopedics department during the COVID-19 pandemic [23]. The authors highlight the importance of establishing criteria for the triaging of patients appropriate for telemedicine visits and quickly assessing the technological resources needed to implement a program. Only 1 of 36 physicians within their department had the resources to begin telemedicine visits at the onset of the pandemic [23]. However, within 2 weeks of initiating services, their orthopedics department was able to reach nearly 50% of their pre-pandemic patient volume. Similarly, this was echoed within our own musculoskeletal service line as none of our providers were prepared to begin telehealth visits at the onset of the COVID-19 non-essential visit discontinuation on March 16, 2020. Our department was also able to achieve 46.8% of normal volume within 3 weeks of initiating our telemedicine program, with that number rising to 61.4% by the fifth week. It should be noted that the volume of orthopedic surgical cases within our department—and across the country—has drastically decreased due to limitations on elective procedures in areas of ongoing COVID-19 transmission [22]. It is also reasonable to assume that given the cancelation of sporting events and other non-essential work, the total volume of orthopedic injuries have decreased [8]. This implies that the clinical volume deficit observed is secondary to the widespread effects of the pandemic.

The roll out of the telemedicine platform to our musculoskeletal service line involved several small work groups charged with developing and overseeing the process. This included weekly training with leadership, physicians, scheduling and patient access, administrative staff, and patient financial services. This resulted in rapid introduction of telemedicine concurrent with implementation of a 2-week alternating schedule for all clinical staff and providers which allowed for preservation of a healthy team with minimal exposure risk, thereby protecting patients and staff while preventing interruption of essential orthopedic care [35]. This schedule was designed to limit staff exposure to 2 concurrent weeks out of 4, respecting the possible incubation period of the virus, while telemedicine was employed during all 4 weeks to allow for social distancing, limit exposure of staff and patients, and allow for continuity of care despite quarantining [35]. Significant improvements in processes occurred following initial implementation to enhance patient experience and quality of care including administrative assistants contacting patients to discuss converting their visit to telemedicine visits, physician best practices for virtual clinic workflow, master schedule template redesigns, creation of a telemedicine platform help line for patients to test connections prior to visits, a team to coach patients on digital upload of outside imaging studies prior to their telemedicine visit, a daily user group forum for the staff and physicians to discuss barriers to implementation, and a team dedicated to telemedicine coding and billing best practices. The importance of these work groups cannot be understated in regard to the successful outcomes of rapid deployment of an efficient, user-friendly, large volume telemedicine presence.

In recent years, orthopedic telemedicine programs have been successfully implemented in the postoperative period of shoulder, hip, and knee surgeries [5,19,32,33,36]. Studies suggest that the use of telemedicine relieves time burden on both physicians and patients but does not diminish the patient’s experience. Kane et al demonstrated the safe use of telemedicine in the postoperative period following rotator cuff repair, showing no difference in patient satisfaction and decreased time for the provider and patient [19]. In addition, they showed that patients had a higher affinity for the platform following the initial experience. Our results and experience mimic the willingness to use telemedicine for future visits, as up to 90% of patients stated they would be willing to utilize telemedicine for another visit (Table 5). Sharareh et al showed no difference in patient-reported satisfaction or clinical outcomes using a telemedicine service for follow-up after total hip and knee arthroplasty [36]. Although our study revealed decreased telemedicine satisfaction overall, Sharareh et al included only postoperative appointments, which are often less time consuming than the average visit; the findings from Sharareh et al may not apply to all types of clinic visits seen in our study and may account for the discrepancy in telemedicine satisfaction. To our knowledge, no orthopedic studies to date have spoken to variation in patient satisfaction with respect to age, though younger patients preferring telemedicine visits is well documented [10,18].

Our data demonstrate a small overall decrease in satisfaction with telemedicine visits compared with in-office visits of 0.27 on a scale of 5 (5.4%) during the COVID-19 pandemic. This finding is in contrast to previously reported studies. While acknowledging the observed decrease in satisfaction is quite small, the authors acknowledge that the difference may have limited clinical relevance. This deviation may be due to a number of factors including mandated use of telemedicine by all non-emergent patients and lack of familiarity with virtual medicine. Unlike the aforementioned studies, we included all clinical visits as opposed to solely postoperative follow-ups. It is reasonable to believe that established patients who fall outside of the postoperative group may be more likely to desire an in-office intervention and would therefore have lower satisfaction. However, on subgroup analysis, our data show no difference in telemedicine satisfaction between new patients and previously established patients. Previous studies have noted that despite randomization, subjects consenting to participate in a telemedicine program were typically younger or more comfortable with technology [10,18]. Our study is unique in that the unforeseen circumstances forced by COVID-19 have revealed an increased need for education and system configuration prior to the telemedicine visit. Our study corroborates multiple studies that have noted an increased level of discomfort using telemedicine products in older populations; however, our data did not show a difference between patient age and satisfaction (Table 5) [19,36].

While our current telemedicine practices were implemented as the result of a once in a generation disaster, it enables us to peer into the utility of telemedicine in musculoskeletal ailments in the future. Multiple studies have demonstrated the time saving benefits of telemedicine for both patients and physicians [16,19,36]. In addition, Vuolio et al found no difference in overall disease management of patients with osteoarthritis at 1-year following the use of telemedicine and in-office visits [39]. Our study shows maintained clinical volume, patient satisfaction, and decreased time burden using telemedicine broadly.

In conclusion, this study demonstrates that a telehealth platform can be rapidly implemented across a large musculoskeletal service line without compromising patient satisfaction or overall experience. The patients agreed across affected body region and age cohort that physicians did not sacrifice overall visit quality. Participants were able to see their regular provider in an efficient and timely fashion during a time when clinical visits would otherwise not be possible. The body of literature exploring safe telemedicine implementation in musculoskeletal care is scarce prior to the COVID-19 pandemic and represents an expanding field for future study. With increasingly busy clinical schedules, creative solutions are needed to meet patient demand, especially when there is likely to be a surge of patient volume when quarantine restrictions are lifted. Patient safety remains paramount, but the use of widespread telehealth has tremendous potential for relieving patient and provider burden without sacrificing quality of care. Further studies are needed to evaluate the long-term clinical implication of this platform and its outcome on accuracy of diagnosis and subsequent patient-reported outcomes during non-pandemic times.

## Supplemental Material

sj-pdf-1-hss-10.1177_1556331620977171 – Supplemental material for Telehealth Can Be Implemented Across a Musculoskeletal Service Line Without Compromising Patient Satisfaction Click here for additional data file. Supplemental material, sj-pdf-1-hss-10.1177_1556331620977171 for Telehealth Can Be Implemented Across a Musculoskeletal Service Line Without Compromising Patient Satisfaction by Samuel A. Taylor, Joseph D. Lamplot, Paul T. Greenfield, Wesley J. Manz, Emily L. DeMaio, Sage H. Duddleston, John W. Xerogeanes, T. Scott Maughon, Corey C. Spencer, Alexander Dawes, Scott D. Boden, Kyle E. Hammond, Eric R. Wagner, Michael B. Gottschalk, Charles A. Daly and Mathew W. Pombo in HSS Journal®: The Musculoskeletal Journal of Hospital for Special Surgery

sj-zip-2-hss-10.1177_1556331620977171 – Supplemental material for Telehealth Can Be Implemented Across a Musculoskeletal Service Line Without Compromising Patient Satisfaction Click here for additional data file. Supplemental material, sj-zip-2-hss-10.1177_1556331620977171 for Telehealth Can Be Implemented Across a Musculoskeletal Service Line Without Compromising Patient Satisfaction by Samuel A. Taylor, Joseph D. Lamplot, Paul T. Greenfield, Wesley J. Manz, Emily L. DeMaio, Sage H. Duddleston, John W. Xerogeanes, T. Scott Maughon, Corey C. Spencer, Alexander Dawes, Scott D. Boden, Kyle E. Hammond, Eric R. Wagner, Michael B. Gottschalk, Charles A. Daly and Mathew W. Pombo in HSS Journal®: The Musculoskeletal Journal of Hospital for Special Surgery
