# Supplementary material for: Telehealth Can Be Implemented Across a Musculoskeletal Service Line Without Compromising Patient Satisfaction
Source: HSS J. 2021 Feb 21;17(1):36–45. doi: 10.1177/1556331620977171 (PMC8077987; doi:10.1177/1556331620977171)
Supplement: sj-pdf-1-hss-10.1177_1556331620977171 – Supplemental material for Telehealth Can Be Implemented Across a Musculoskeletal Service Line Without Compromising Patient Satisfaction [file sj-pdf-1-hss-10.1177_1556331620977171.pdf]

1. How satisfied were you with your last in-office visit?
    a. Very satisfied
    b. Satisfied
    c. Neutral
    d. Dissatisfied
    e. Very dissatisfied
2. How much time did this in-office visit require (in minutes) including travel?
3. How satisfied were you with your telemedicine visit?
    a. Very satisfied
    b. Satisfied
    c. Neutral
    d. Dissatisfied
    e. Very dissatisfied
4. How much time did this telemedicine visit require (in minutes) including set up?
5. Would you use telemedicine for a visit in the future?
    a. Yes
    b. No
    c. No preference
6. Would you have preferred to have in-office or telemedicine visits?
    a. In-office
    b. Telemedicine
    c. No preference
7. How would you rate the ease-of-use in setting up/utilizing the telemedicine platform?
    a. Very difficult
    b. Somewhat difficult
    c. Neutral
    d. Somewhat easy
    e. Very easy
8. On a scale of 1-5, please rate the following:*
    a. Physician's communication:
    b. Physician's physical examination:
    c. Physician's explanations of plan/options:
    d. The amout of time you had to speak with the physician:
9. How would you rate your current pain level (VAS) ǂ
10. Did you require assistance to get to your appointment/set up the telemedicine appointment? (Yes/No)
11. Did this visit require you to take off of work?
    a. Yes
    b. No
    c. N/A

*Associated scale values are as follows: 1 - very poor, 2 - poor, 3 - neurtal, 4 - good, 5 - very good

ǂStandard 0-10 VAS pain scale was used
